# 4D Printing Shape-Morphing Hybrid Biomaterials for Advanced Bioengineering Applications

**DOI:** 10.3390/ma16206661

**Published:** 2023-10-12

**Authors:** Irene Chiesa, Maria Rachele Ceccarini, Silvia Bittolo Bon, Michela Codini, Tommaso Beccari, Luca Valentini, Carmelo De Maria

**Affiliations:** 1 Department of Ingegneria dell’Informazione and Research Center E. Piaggio, University of Pisa, Largo Lucio Lazzarino 1, 56122 Pisa, Italy; irene.chiesa@ing.unipi.it; 2 Department of Pharmaceutical Sciences, University of Perugia, 06123 Perugia, Italy; mariarachele.ceccarini@unipg.it (M.R.C.); michela.codini@unipg.it (M.C.); tommaso.beccari@unipg.it (T.B.); 3 Physics and Geology Department, University of Perugia, Via Pascoli, 06123 Perugia, Italy; silvia.bittolo@gmail.com; 4 Civil and Environmental Engineering Department, University of Perugia, Strada di Pentima 4, 05100 Terni, Italy; luca.valentini@unipg.it

**Keywords:** 4D printing, biomaterials, functional materials, bioengineering

## Abstract

Four-dimensional (4D) printing is an innovative additive manufacturing technology used to fabricate structures that can evolve over time when exposed to a predefined environmental stimulus. 4D printed objects are no longer static objects but programmable active structures that accomplish their functions thanks to a change over time in their physical/chemical properties that usually displays macroscopically as a shapeshifting in response to an external stimulus. 4D printing is characterized by several entangled features (e.g., involved material(s), structure geometry, and applied stimulus entities) that need to be carefully coupled to obtain a favorable fabrication and a functioning structure. Overall, the integration of micro-/nanofabrication methods of biomaterials with nanomaterials represents a promising approach for the development of advanced materials. The ability to construct complex and multifunctional triggerable structures capable of being activated allows for the control of biomedical device activity, reducing the need for invasive interventions. Such advancements provide new tools to biomedical engineers and clinicians to design dynamically actuated implantable devices. In this context, the aim of this review is to demonstrate the potential of 4D printing as an enabling manufacturing technology to code the environmentally triggered physical evolution of structures and devices of biomedical interest.

## 1. Introduction

Additive manufacturing (AM) technologies (also known as “three-dimensional (3D) printing”) have been widely exploited in many interdisciplinary research fields (such as automotive, soft electronics, and bioengineering) due to their excellent repeatability and capacity to construct complex structures with precise geometry control [1,2]. AM processes start from a digital model, designed by computer-aided design (CAD) software or obtained by segmentation of surfaces or tomographic scanning data, to fabricate the desired structure layer-by-layer, i.e., adding successive layers of materials onto previously deposited/solidified ones [3].

In the last decades, different innovations have been introduced in AM, such as multi-material and multi-scale AM, in which different AM technologies are combined to fabricate heterogeneous structures [4,5], in situ printing, in which material deposition occurs on non-planar complex surfaces [6,7], and four-dimensional (4D) printing, in which active structures are designed and manufactured [8,9].

More in detail, 4D printing was introduced in 2013 by Dr. Skyler Tibbits [10] to denote the fabrication via AM of structures with the capability to shape transform over time, which represents the “fourth dimension”, under a predefined stimulus. Some of the characteristics most frequently associated with 4D printed structures are shape-changing, self-repairing, and self-assembly, emphasizing that these are no longer static objects but rather programmable active structures that carry out their function by changing their physical and/or chemical properties over time in response to a predetermined stimulus [11,12].

Therefore, the key points of 4D printing are: (i) active materials (also known as smart materials or stimuli responsive materials) or combinations of different materials; (ii) external stimuli (e.g., temperature, humidity, electric stimulation, pH, and light); and (iii) AM technologies, which act as an enabling tool that allows the exact positioning of a precise quantity of one or more stimulus-responsive materials in predetermined locations without restrictions on the complexity of the geometry [9,13,14]. The deep description of the different AM technologies is not in the scope of this review. If the reader is interested in this topic, we suggest the following reviews as seminal works in the field: [15,16].

Using two dimensional (2D) nanomaterials (e.g., graphene), 4D-printed nanocomposites have been used to design shape-programming objects. For example, Wei et al. [17] used 4D printing to obtain acrylonitrile–butadiene–styrene/poly(lactic acid)/graphene composites that had a linear thermal coefficient of 75 ppm°C^−1^. Moreover, laser printing technology has been used in synergy with 4D printing to convert graphene oxide into graphene-based composites [18]. This laser technique can be applied to induce the formation of graphene on biodegradable substrates [19,20].

Using responsive materials and the related stimuli for their activation, it is possible to physically program many morphological transformations enabled by the proper organization guaranteed by AM. However, designing a new structure via 4D printing is a complex problem. Indeed, it is influenced by several variables (e.g., stimulus, materials, geometries, mechanisms of interactions) that need to be properly combined to achieve a functional structure. Moreover, the knowledge of the material’s behavior and interactions, the correct stimulus, and the printing parameters are fundamental elements that increase the complexity of the problem and that need to be considered and deeply understood. In this context, mathematical modeling is a very useful tool to determine the combination of variables that leads to the maximum and desired transformation of the 4D-printed structures [21,22].

Since 2013, when 4D printing was introduced, this fabrication strategy has seen fast growth in many sectors, including smart textiles, autonomous and soft robotics, biomedical devices, electronics, and tissue engineering (TE). This is due to its advantages over static AM, such as the possibility of an easier fabrication of complex 3D structures that are fabricated as flatted objects and then achieve their 3D conformation after actuation, thus reducing the encumbrance of the objects, and the exploitation of reliable alternatives to electrical energy (e.g., chemical potential, elastic energy), capable of remote control without the use of cumbersome wiring, thus leading to the use of 4D printed structures in severe environments (e.g., human body, low-resource settings).

In this review, we aim at providing an overview of 4D printing as a new additive manufacturing technology in the biomedical field. In the first part, a brief introduction to stimuli-responsive materials with a special focus on natural polymers and nanomaterials will be provided, along with a description of the achievable transformations and mathematical modeling. Then, applications of 4D printing in bioengineering (e.g., soft actuator fabrication, medical device design, TE, and drug delivery) will be discussed, reporting relevant examples from the recent literature.

## 2. Stimuli-Responsive Materials in 4D Printing

In many applications, 4D printing exploits active materials, namely materials that undergo useful, predictive, reproducible, and macroscopic physical or chemical changes in their properties as a consequence of an environmental change [2,23]. While there are examples of active materials in all material classes (mostly metals, polymers, and ceramics), smart polymers (such as shape memory polymers (SMPs) and liquid crystal elastomers) have been favored for 4D printing due to their easy processing and wide range of stimuli that they may be used with. Smart polymers could be activated by a variety of external stimuli, including temperature, pH, electric field, magnetic field, and light.

With the rapid progress of 4D printing, the prevalence of eco-friendly, sustainable smart polymers from natural sources is gaining great interest in the field [24,25,26]. The exploitation of natural polymers is prompted by the fact that they possess enhanced biocompatibility and bioactivity if compared with synthetic materials, which makes it easier for natural polymers to be interfaced with biological systems, including living cells [27]. Biocompatibility is a crucial point in biomedical applications to guarantee the safety of patients and the vitality of cells in vitro, which currently limit the choice of materials in 4D printing [28]. It is important to highlight that biocompatibility must also be applied to the stimulus that is used to trigger the shape-morphing of the structures. Indeed, the materials must react to an environmental variation that is in accordance with the target application. For example, if the structure is designed to interface with human cells, the materials, in addition to being biocompatible, must react to a stimulus compatible with cell wellness (e.g., temperature between 20 °C and 37 °C, pH around 7.4).

Moreover, exploiting natural biomaterials allows for the expansion of the library of possible bioinks/biomaterial inks, thanks to the variety of natural building blocks, such as peptides, amino acids, and deoxyribonucleic acid (DNA) sequences.

In the following paragraphs, the active polymers, with a deep focus on natural polymers, used in 4D printing will be analyzed and classified in relation to the involved stimulus.

### 2.1. Temperature-Responsive Materials

Currently, one of the most investigated methods to achieve shape-changing in 4D printing is temperature responsiveness. A thermo-responsive material can employ exogenous temperature changes as stimulus to achieve a particular shape transformation or can be activated by heating through the Joule effect due to electrical current flow. The easiest form of temperature-responsiveness is thermal expansion, which leads to the volume expansion of a structure as a consequence of a temperature increase. In this context, there are also a few materials that exhibit the opposite behavior, undergoing contraction with an increase in temperature [29]. Among them, silk fibroin (SF) attracted a lot of attention from researchers because of its extracellular matrix (ECM)-likeness, low cost, adjustable mechanical properties, controllable degradation, and good biocompatibility [30]. Moreover, the timeline of the development of SF-based ink in 3D printing technology over the past 30 years has witnessed great research and application value for the customized biomedical field [31]. These results encouraged further exploration of SF-based biomaterials via 4D printing.

Taking a step forward, changes in temperature can induce variations in wettability and solubility alterations of materials, as for poly(N-isopropylacrylamide) (PNIPAAm), poly(methyl vinyl ether), and poly(N,N-dimethylaminoethyl methacrylate) [22,32].

### 2.2. Humidity-Responsive Materials

An easy method that fosters the temporal shape transition of 4D-printed structures is humidity responsiveness. The phenomenon refers to the inherent swelling feature observed in both synthetic and natural hydrogels [33]. Hydrogels are 3D cross-linked polymeric networks that can absorb and hold massive quantities of water [34,35]. Their ability to absorb water without dissolving in a thermodynamically favorable solvent can be referred to as their swelling ability. This is due to their chemically or physically cross-linked network, which experiences a reversible volume change when dipped in a suitable solution [36]. Gelatin and collagen, among other hydrophilic natural polymers, have been utilized in 4D printing as humidity-responsive materials [37].

### 2.3. pH-Responsive Materials

Another category of active materials are pH-responsive polymers. They can vary their rheological characteristics, such as viscosity and shear modulus, in response to changes in the pH or ion concentration of the surrounding environment [2,23,38]. Either the protonation of ionizable groups or the deterioration of acid-cleavable bonds can be responsible for this phenomenon. In more detail, the polymeric chains of those materials can stretch to a coil form as a result of the charged functional groups’ electrostatic repulsion or form globule structures when the charge of the functional groups is neutralized [39]. Collagen, gelatin, and keratin are only a few examples of naturally occurring polymers that, exhibiting pH responsiveness, undergo a change in their swelling/shrinking profiles in response to various pH environments [23,40].

Moreover, pH can be utilized to promote the self-assembly of peptide hydrogels made of the alternation of natural amino acids, increasing the mechanical and rheological properties of the substance by promoting the creation of intra-molecular sheets or alpha-helices [41,42].

### 2.4. Light-Responsive Materials

In light-sensitive materials, the applied optical stimulation (e.g., visible, ultraviolet (UV), and near-infrared (NIR) light) is converted into other responses, usually mechanical ones [43]. The chromophore that is included in the materials will determine whether a light-responsive activity is reversible or irreversible. The most commonly used chromophores are photochromic compounds (such as azobenzene, spiropyran, and salicylideneaniline) that change polarity and go through isomerization when exposed to light [44,45]. Light has distinct advantages over other stimuli, such as a spatially controlled activation region that may be achieved by using a photomask or focused light source, as well as an instant activation that is simple to stop, pause, and resume [46]. Due to their exceptional optical responsiveness, nanomaterials like carbon nanotubes and materials based on graphene have recently been employed as light-sensitive components [47]. 

It is crucial to emphasize that in numerous works, light is employed as a substitute technique to heat the 4D structure in specific spots. Consequently, the rise in temperature brought on by the lights is the true stimulus that causes the shape-shifting [48,49].

### 2.5. Electric Field Responsive Materials

Polythiophene and poly(2-hydroxyethyl methacrylate) are some examples of electrically responsive materials characterized by intrinsic electrical conductivity. Their shape and size can be regulated by the intensity and direction of an external electric field [50,51].

Coulombic, electrophoretic, electroosmotic, and piezoelectric processes are some examples of the electrical interactions that can take place in a material to produce electric field responsiveness [52]. Additionally, passive polymers and electro-sensitive particles, such as dielectric polarizable particles, can be combined to create electrically responsive materials [53]. These particles polarize when exposed to an electric field, altering the material structure [53].

### 2.6. Magnetic Field Responsive Materials

Magnetic fields, in addition to electrical fields, are frequently utilized for triggering shape changes in 4D-printed structures. Uncontactable remote manipulation using magnetic fields is effective and safe [23]. Similar to electrical responsive materials, magnetic responsive materials typically contain uniformly dispersed magnetic-sensitive particles (such as cobalt ferrite, iron platinum, and iron oxide) in a carrier solution [52,53].

### 2.7. Shape Memory Polymers

SMPs are a widely utilized class of smart polymers that are crucial for 4D printing in the biomedical industry [23,24]. Their smart behavior could be activated by different types of stimuli, such as temperature, water, or an electric field [54]. When SMPs are exploited, a programming phase is necessary in which the material/structure is manually deformed by exposure to the triggering stimulus. Thus, a temporary shape is generated and subsequently fixed by the quick removal of the stimulus. Then, the stressed polymer relaxes as a result of an additional exposure to the triggering stimulus, enabling the structure to regain its original shape [21].

Polylactic acid (PLA) and acrylonitrile butadiene styrene (ABS) are the most commonly used temperature-responsive SMPs [24,25], whereas polyurethane and poly(butanetetrol fumarate) are the major representatives of water-responsive SMPs [55,56].

## 3. Achievable 4D Transformations

In the literature, there are different attempts to define a taxonomy of shape-changing structures that can be obtained with 4D printing. Here, starting from the classification provided by Nam and Pei [57], an advanced library of programmable transformations is defined (Table 1).

## 4. Mathematical Modeling for 4D Printing

Four-dimensional printing is influenced by several entangled variables, such as the entity of the applied stimulus, the spatial deposition of different material(s), and their properties. Those variables need to be carefully coupled to obtain a favorable fabrication and a functioning structure, making the design of a new structure via 4D printing a complex problem. In this contest, the development of an appropriate mathematical model is essential for the success of the 4D printing process [78,79,80]. Mathematical models allow for the prediction of the shape evolution of the structure over time, thus avoiding eventual collisions between the components, facilitating the achievement of the desired movements, and reducing the copious and time-consuming trial-and-error experiments that usually characterize the development of a new device/technology.

An appropriate mathematical model for 4D printing comprises four main elements: (i) the final desired shape (e.g., desired bending or twisting angles), dictated by the application of the 4D printed object; (ii) the spatial deposition of the involved material(s), i.e., the initial shape. This aspect closely depends on the employed AM technology and on the material processability; (iii) material properties and responsiveness to the applied stimulus; and (iv) entities of the applied stimulus.

Mathematical models can be divided into two main categories: forward problems and inverse problems. The forward problems aim at determining the final shape of the structure and are more oriented toward discovering concepts. A glaring example of a forward problem is the use of lumped parameter models to study the shape evolution of SMPs. For instance, the standard linear solid model (i.e., a parallel arrangement consisting of one elastic spring and one Maxwell element) was exploited by Yu et al. to quantitatively model the energy storage and release process achieved during the multiple shape transformation in SMPs [81].

In inverse problems, the final state is known, and the user aims to find out how to obtain it by defining the initial geometries and spatial deposition of material(s) (i.e., the printing path). Thus, inverse problems are more application-oriented. A brilliant example of an inverse problem can be found in the study of Gladman et al. [61], in which the authors identified the print path required to mimic the complex curvature of the calla lily flower.

Analytical models represent the mathematical formulation of a certain mechanical or biophysical system or phenomenon, thus being extremely used in the mathematical modeling of 4D printing [82]. Examples of analytical models in 4D printing are the beam and plate theories (e.g., Euler–Bernoulli model and Timoshenko’s model) [83], or the spring-mass systems (e.g., Maxwell system, standard linear solid) [84]. In the case of complex geometries and/or motions, analytical models may not have a closed-form solution, so numerical analyses are required. Consequently, mathematical models are implemented via computational models that exploit computers to study and simulate complex systems that could involve highly deformable bodies as well as multiscale and multiphasic features. Examples of computational models for 4D printing could either rely on the Mass-Spring System (MSS) or on Finite Element Modeling (FEM) [85,86,87].

## 5. 4D Printing in the Biomedical Field

### 5.1. Bioactuators

Soft actuators, also referred to as bioactuators, are highly deformable structures characterized by ease of movement that can be activated by external stimuli to generate the desired motion and forces/torques [88]. They are usually constituted by materials with low elastic moduli (e.g., silicone, electroactive polymers) or fluids, thus the designation “soft” actuators [89]. They present several key advantages compared to traditional rigid actuators: (i) the possibility of miniaturization; (ii) a few components; (iii) actuation through low-power external stimuli; (iv) deformability and complex motion; and (v) the ability to mimic the softness and body compliance of biological systems [88,90,91]. AM technologies have been used in several studies to fabricate soft actuators [92,93]. However, the actuation is usually not intrinsic to the printed object but obtained by external fluids or compressed air. In this scenario, 4D printing could offer the tools to simplify the fabrication of soft actuators with the intrinsic capability to self-actuate under a precise stimulus, thus obviating the need for external actuation [92]. Moreover, 4D printing can significantly reduce the fabrication cost of soft actuators thanks to the possibility of integrating their different parts through the stimulus-triggered self-assembly of the structure itself, thus reducing the need for strict tolerances [93,94].

A milestone example of this use of 4D printing for soft actuators is provided by Liu et al. [95]. The authors combined a high swelling (i.e., active) and a low swelling (i.e., passive) material in tubular geometries to achieve several different movements, such as uniaxial elongation, radial expansion, bending, and gripping, to develop a new soft actuator. In more detail, inspired by coral polyps, the authors designed and 4D printed tubes with self-folding fingers at one end, exploiting extrusion-based 3D bioprinting (EBB). PNIPAAm was used as an active thermo-responsive material and simultaneously deposited with polyacrylamide (PAAM), used as a passive support material. In this way, the structures exhibit both uniaxial expansion of the tube and finger gripping when dipped in water at a temperature higher than 35 °C.

Taking a step forward, Chan et al. [96] fabricated a biological machine with an actuation module for locomotion that was produced by a cluster of viable cardiomyocytes. More in detail, the authors first fabricated the main structure, comprising a cantilever and a base in poly(ethylene glycol) diacrylate (PEGDA), by stereolithography (SLA). Then, the cantilever structure was seeded with a monolayer of contractile cardiomyocytes. This biological machine exhibited a walking motion with a maximum speed of 236 μms^−1^, thus possessing a highly efficient mechanism of locomotion. This was achieved using the cell contractive forces and the anisotropic friction of the supporting structure.

### 5.2. Tissue Engineering

Native tissues exhibit complex 3D structures and microarchitectures, as well as unique functions that are achieved through dynamic changes in tissue conformation [62]. Therefore, due to their static behaviors, 3D bioprinted scaffolds are not able to closely recapitulate native tissue dynamics. By creating physiologically active scaffolds that can change their shapes in response to desired stimulation, 4D printing could potentially overcome this difficulty and emulate the movements of the actual tissue [97]. In this context, 4D printing can be referred to as 4D bioprinting, and the biocompatibility of both materials and stimulus is a crucial point because it must be ensured that the scaffold materials support cell growth and tissue development without causing harm [98,99]. For this reason, the biocompatibility of a 4D-bioprinted scaffold should be evaluated not only in vitro (cell culture) but also in vivo. Moreover, in some cases, 4D-printed objects may incorporate living cells or other biological agents. Ensuring the biocompatibility of these components is vital to their integration and function within the body.

The 4D-bioprinted scaffolds can improve the functionality of the scaffold by simplifying its seeding and the fabrication of structures capable of adapting to the complex 3D shapes of the human body or providing appropriate stimuli to promote cell differentiation and activities [100,101].

For example, in the context of bone TE, it is difficult for traditional 3D static scaffolds to have a good match with the sharp edge shape of bone defects, thus often leading to incomplete edge and material absorption [102,103]. In this context, 4D bioprinting can provide a solid solution that possesses great advantages in minimally invasive surgery and defect shape matching. With this aim, Shuai et al. [56] exploited the water-triggered shape memory effect of thermoplastic polyurethane to fabricate smart scaffolds for bone defect repair. More in detail, the scaffolds, fabricated via selective laser sintering, were able to be compressed up to 67% when pre-immersed in deionized water and to maintain this temporary shape after drying. Then, when they were re-immersed in water, the shape recovery ratio reached 90%. In vitro biocompatibility tests showed that the shape-recovered scaffold could promote cell adhesion and direct cell proliferation.

**Figure 1:**
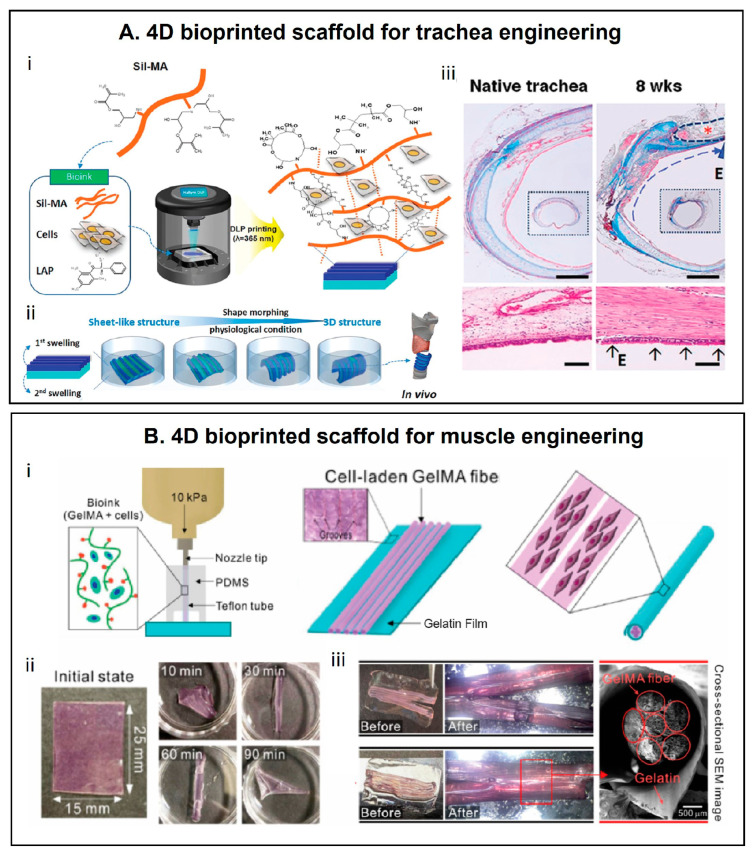
(**A**) 4D bioprinted trachea scaffold developed by Kim et al. [62]. (**i**) Chemical structure of Sil-MA used to fabricate cell-laden 4D shape morphing structures by DLP. (**ii**) Self-shape morphing behavior of the Sil-MA bilayer scaffold in water due to differential swelling. (**iii**) Masson’s trichrome staining of the native trachea and trachea treated with the 4D bioprinted constructs after 8 weeks of surgery (scale: 1 mm; 1 cm for images in small boxes), and histological staining revealing newly formed respiratory epithelium 2 weeks after the implantation (scale: 0.1 mm). The engineered trachea is marked with asterisks and dotted line. Newly formed respiratory epithelium was marked as E and short arrow. Figure reproduced with permission from [62]. (**B**) 4D bioprinted skeleton muscle scaffold developed by Yang et al. [63] (**i**) Schematic of the fabrication and actuation of the constructs. (**ii**) Swelling-driven self-folding ability of gelatin films. (**iii**) Images of the real constructs before and after actuation. Scanning emission microscopy images confirmed the bundle-like structure of the constructs. Figure reproduced with permission from [63].

Moving to a diverse anatomical district, Kim et al. [62] 4D-bioprinted via digital light processing (DLP) an in vitro trachea scaffold, exploiting methacrylated silk (Sil-MA) (Figure 1A(i)). The authors fabricated a bilayer Sil-MA scaffold with different Sil-MA concentrations in each layer, thus exploiting the differential swelling properties of the two layers to obtain the self-folding of the structure, thus resembling the architecture of native trachea (Figure 1A(ii)). Moreover, to mimic the heterogeneity of the native traches, two types of cells were introduced in the layers: turbinate-derived mesenchymal stem cells (TBSCs) were added in the bioink of the base layer, whereas human chondrocytes were included in the bioink in the pattered layer, thus mimicking the hyaline cartilage ring of the native trachea. After the manufacturing, the cell-laden bilayer structure was immersed in culture medium and incubated to induce self-folding to the planned morphology. Preliminary in vitro and in vivo studies revealed that the 4D-printed scaffold is highly biocompatible and underwent stable integration with the host trachea, showing regeneration performances (Figure 1A(iii)).

Focusing on the same tissue (e.g., cartilage) and shape-morphing strategies, Ding et al. [104] fabricated a bilayer scaffold made of cell-laden oxidized and methacrylated alginate for cartilage TE. More in detail, the scaffold possesses a crosslinking gradient that provides to the structure a differential swelling behavior, which in turn leads to the self-folding over time. After the actuation in culture media, the scaffold obtains a C-like shape that leads to the condensation of the cells cultured in the inner layer, which shows a high vitality and the production of glycosaminoglycans comparable with static control.

Taking a step forward, Yang et al. [63] fabricated a 4D bioprinted construct able to mimic the complex structure of the perimysium of a parallel fusiform skeleton muscle, exploiting the swelling capabilities of gelatin films (Figure 1B(i)). In more detail, firstly, the authors fabricate via EBB gelatin films with grooves on their top. The grooves, acting as hinges, enable the folding of the gelatin films once dipped in water-based solutions (Figure 1B(ii)). Then, cell-laden methacrylaed gelatin (gelMA) fibers were deposited via EBB on the gelatin film. Thus, when this planar construct is dipped in culture medium and the gelatin film self-folds, a bundled structure comprising the cell-embedded microfibers on its inside is created (Figure 1B(iii)).

Finally, thinking about patient-adaptable soft tissues such as blood vessels, Luo and colleagues created an effective approach to manufacturing scaffolds with fine topologies combining 4D printing, fused deposition modeling (FDM), and crosslinkable shape memory linear copolyesters [105]. These 4D scaffolds demonstrated excellent biocompatibility, and under UV-assisted irradiation, they showed incredible shape memory recovery, optical mechanical performance, and good stability in a water environment.

### 5.3. Medical Devices

A medical device is any article manufactured to be used in human beings with the final aim of diagnosis, prevention, monitoring, prediction, prognosis, treatment, or alleviation of disease, injury, or disability, as proposed by the Global Harmonization Task Force and reported in official legislation as European Regulation 745/2017 [106,107]. 4D printing has the potential to drive a significant transformation in the medical devices field due to its potential capability to manufacture: (i) customized implants able to grow up with human growth; (ii) devices that allow the use of minimally invasive surgical procedures; and (iii) active devices that perform their action without the need for electrical energy [108]. For example, Lin et al. [109] developed a 4D-printed adsorbable left atrial appendage occluder (LAAO), a medical device that aims at reducing the risk of left atrial appendage blood clots entering the bloodstream, thus reducing stroke occurrence. More in detail, the device was fabricated via FDM using magnetic nanocomposite-laden PLA that possesses shape memory properties. The addition of the magnetic nanoparticles ensured the self-heating and the remote-control of the device 4D transformation. The 4D-printed LAAO was programmed to a straight temporary shape with a small cross-section area to facilitate interventional delivery and implantation. Then, upon heating, the structure opens, coming back to its permanent open shape, thus performing its occluding function.

**Figure 2:**
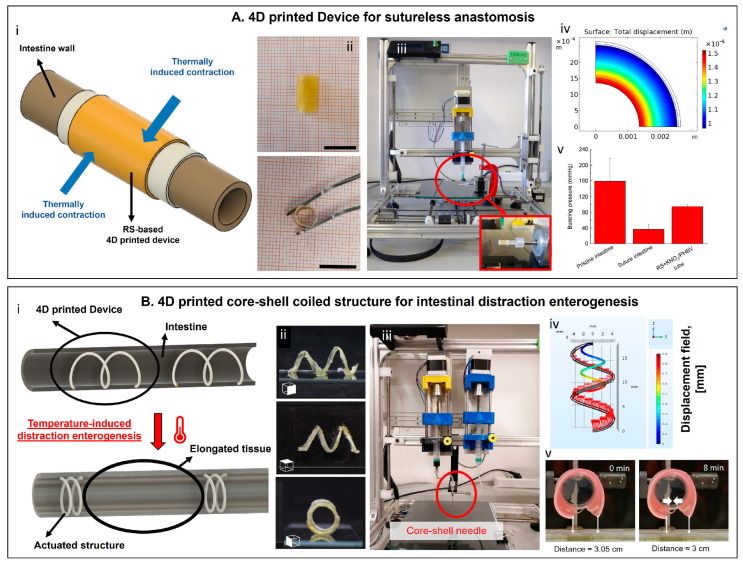
(**A**) Four-dimensional-printed clips for intestinal anastomosis. (**i**) Rationale of the device; (**ii**) photo of the bilayer clips, scale bar = 1 cm. (**iii**) An EBB equipped with a rotating spindle is used in the work. (**iv**) Finite element simulation, showing the ability of the clips to contract and compress the intestine wall with an increase in temperature. (**v**) Validation of the device through ex vivo tests. Figure reproduced with permission from [58]. (**B**) Four-dimensional-printed core-shell coiled structure for intestinal distraction enterogenesis. (**i**) Rationale of the device; (**ii**) photo of the core-shell springs. (**iii**) An extrusion-based 3D printer equipped with a core-shell needle is used in the work. (**iv**) Finite element simulation, showing the ability of the springs to torque and compress with the increase in temperature. (**v**) Validation of the device through phantom tests. Figure reproduced with permission from [59].

In previous works, we exploited the temperature responsiveness of regenerated silk (RS) for the fabrication of two devices to be used in intestinal surgery applications [58,59]. The same combination of active (i.e., RS) and passive (i.e., poly(3-hydroxybutyrate-co-3-hydroxyvalerate)) materials was used, as well as the same stimulus (i.e., the increase of temperature from the room temperature to the body temperature). The different required behaviors, dictated by the different applications (i.e., intestinal anastomosis and intestinal distraction enterogenesis), were achieved thanks to a different spatial arrangement of the involved materials into bilayer tubes (Figure 2A(i,ii)) and core-shell coiled structures (Figure 2B(i,ii)), respectively. This design freedom and customization were made possible by the use of advanced additive manufacturing technologies, namely EBB equipped with a rotating spindle (Figure 2A(iii)) and a core-shell system integrated into a EBB alongside gel-in-gel deposition strategies (Figure 2B(iii)). FEMs were exploited in both studies to investigate the temperature-triggered contraction of the devices according to the RS-based solution content (Figure 2A(iv),B(iv)). The devices were then validated experimentally. Briefly, the clips for sutureless anastomosis were tested ex vivo on a porcine intestine and were able to withstand a bursting pressure approximately 140% higher than the one registered for conventional sutured samples (Figure 2A(v)). Differently, the core-shell coils for distraction enterogenesis were tested on a porcine phantom and were able to pull the flaps of the phantom closer as a consequence of an increase in temperature (Figure 2B(v)).

### 5.4. Drug Delivery

Drug delivery systems play a critical role in the pharmaceutical industry by ensuring that medications are administered to patients effectively and with precise control. 4D printing can also be applied to drug delivery systems, where printed structures release drugs in a controlled manner. In this context, 4D printing possesses several advantages if compared with traditional manufacturing techniques (e.g., capsule filling and tableting). Indeed, 4D printing offers a number of benefits, including the possibility to obtain a controllable kinetic and to achieve a time- and/or site-dependent drug release based on the shape-shifting property of the device itself [110,111].

4D-printed structures for drug delivery systems should be biocompatible to avoid toxicity or irritation at the delivery site. In this case, 4D-printed drug delivery systems utilize biocompatible hydrogels or smart polymers that can change their properties in response to environmental factors. These materials are well-suited for controlled drug release applications. For example, a chitosan-pectin hydrogel was utilized by Long and colleagues to print a biodegradable wound dressing for the local anaesthetic medication lidocaine [112]. The apparatus functioned as a standard swollen polymeric system that sorbs the solvent and desorbs the loaded medication, thus dissolving the polymeric matrix.

In smart drug delivery system fabrication, temperature-responsive materials are carefully chosen based on their ability to undergo a phase transition, typically a sol-gel transition, within a specific temperature range. Common materials include thermosensitive polymers like PNIPAAm and its derivatives [112]. The drug to be delivered could be encapsulated within the temperature-responsive material during the 4D printing process [113]. This can be achieved by mixing the drug with the polymer solution before printing or by incorporating drug-loaded microspheres or nanoparticles within the printed structure [114]. A fantastic result was recently obtained by Suryavanshi and co-workers who synthesized a novel thermo-responsive self-folding feedstock able to carry paracetamol, a common drug, with extraordinary efficiency [115]. In fact, an in vitro study confirmed that this programmed 4D printer had temperature-responsive shrinkage/swelling properties and was able to release almost 100% of the drug into the gastric pH medium within 4 h.

Moreover, 4D-printed hydrogels with antimicrobial properties have been receiving attention in the last decade, especially for wound closure. 4D hydrogels were functionalized with the cell-adhesive motif Arginine-Glycine-Asparagine (RGD) to enhance cell spreading [116], and at the same time, they may show a strong antimicrobic effect against *Staphylococcus aureus* and *Enterococcus faecalis* [117]. In fact, cells that recognize the RGD motifs can bind to them via the sites of integrin on the cell membrane and proliferate. This process could also be used for drug delivery in a local release region in an intelligent manner, such as dental pulp [118]. Regenerative strategies for endodontics and periodontics have received special attention recently [119,120].

Other examples were represented by 4D-printed drug-eluting stents that could release medications gradually to prevent restenosis in blood vessels [121].

**Figure 3:**
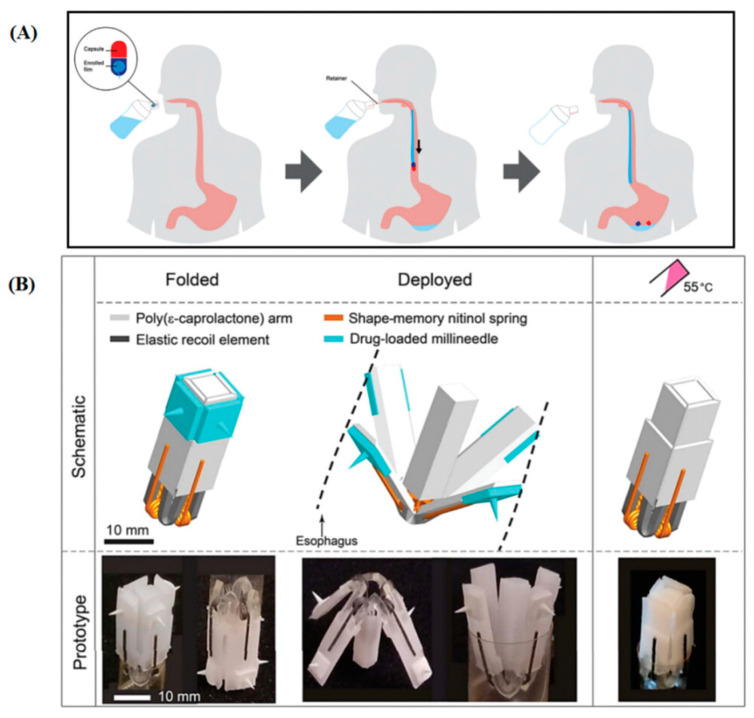
(**A**) Schematic of the application of EsoCap, which is composed of a hard gelatin capsule that contains a sinker to decrease buoyancy and a mucoadhesive enrolled film and retainer thread, using the 3D printed applicator. (**B**) A schematic presentation of the flower-shaped esophageal drug delivery system illustrates its composition, the folded configuration prior to administration, its deployment upon reaching the esophagus, and the recovery of its original shape upon thermal triggering of nitinol wires [121].

Last, but not least, 4D printing enables the fabrication of patient-specific drug delivery systems (Figure 3). Each patient’s unique needs can be considered, and personalized drug dosages or release patterns can be achieved. This is particularly relevant in oncology, where personalized chemotherapy delivery can reduce side effects and improve treatment efficacy [122].

Utilize 4D-printed materials to create drug delivery systems that can release chemotherapy agents with precise control by using the right quantity of the drug at the right time. One example is the prosthesis of paclitaxel and doxorubicin microspheres to prevent tumor recurrence and metastasis after breast-conserving surgery designed and prepared by Hao et al. in 2021 [123].

Other examples were studied to implement real-time monitoring of treatment outcomes and patient responses to adjust drug delivery parameters as needed [124].

For sure, to allow the use of 4D printing in clinical practice in an effectively integrated manner, we must create multidisciplinary care teams involving oncologists, pharmacists, nurses, and 4D printing specialists to facilitate patient-specific treatment planning and implementation.

Finally, by designing drug delivery systems that release medication over extended periods, patients may require fewer doses, improving compliance and reducing the risk of medication errors. The potential for personalized drug delivery through 4D printing brings undoubtedly with it a host of ethical and regulatory challenges. Addressing these challenges is crucial to ensuring the safe and responsible use of this technology. First of all, personalized drug delivery may require the collection and storage of sensitive patient data, such as medical history and genetic information. For this reason, patients must understand and consent to the use of their data and the personalized drug delivery approach. On the other hand, one must implement strict data protection measures, secure storage and encryption, and ensure compliance with data privacy laws (e.g., the General Data Protection Regulation (GDPR) or the Healthcare Insurance Portability and Accountability Act (HIPAA)).

## 6. Conclusions

In conclusion, although several open challenges still must be faced (e.g., development of accurate mathematical models and deep investigation of biocompatible active materials), 4D (bio)printing is a breakthrough technology that, thanks to the constant progress in materials science, additive manufacturing, and biology, represents an enabling tool to address unsolved clinical needs in tissue engineering and medical device manufacturing. This review highlighted the importance and potentiality of 4D (bio)printing to tailor and customize the functionalities of a device by combining active materials with appropriate 3D architectures and advanced AM technologies.

## Figures and Tables

**Table 1:** Classification of the most performed transformations in 4D printing. Images were adapted with permission from [57].

Taxonomy	Description	Schematic Image	Refs.
Expansion and contraction	Description: changing in length, volume, and area. How to achieve: linear swelling and shrinking of thermo-responsive materials after immersion in cold and hot water.	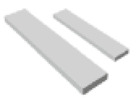	[58,59]
Bending	Description: curvature of the entire structure. How to achieve: swelling/shrinkage mismatch between layered materials.	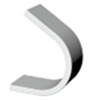	[60,61]
Folding	Description: sharp curvature along a crease on the construct. How to achieve: caused by a stress mismatch between rigid and active materials.	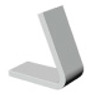	[62,63]
Rolling	Description: the structure moves by turning over and over on its own axis. Materials maintain the same orientation in the deformation direction. How to achieve: usually triggered by heat in the common shape memory cycles that foresee programming and recovery steps.	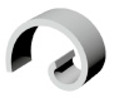	[64,65]
Helixing	Description: a curve traced on a cylinder by the rotation of the structure with a constant oblique angle. How to achieve: can be programmed to perform different deposition patterns and exploit the swelling/shrinkage mismatch between materials.	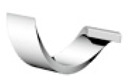	[12,66]
Twisting	Description: curvature created by a rotation of the structure around a stationary point. How to achieve: twisting can be programmed to perform different deposition patterns and exploit the swelling/shrinkage mismatch between materials.	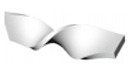	[64,67]
Curving	Description: self-curving occurs when a flat structure performs local deviations from a plane. How to achieve: this activation can be programmed by exploiting the shear stresses at the interface between two different materials disposed in concentric circles.	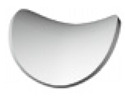	[61,67]
Waving	Description: shape that has regular undulating features or a regular wavy up-and-down form. How to achieve: structures composed of three materials: two active materials inside a passive matrix. In function of the deposition pattern of the two active materials, different waving structures can be fabricated.	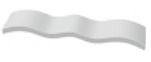	[68,69]
Curling	Description: shape that has irregular undulating features or an irregular wavy up-and-down form. How to achieve: structures composed of three materials: two active materials inside a passive matrix. The curling occurs due to the mismatch in swelling properties between passive and active materials.	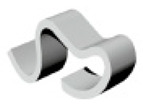	[70,71]
Hierarchical structures	Description: assembling of different active elements and mechanisms that react, resulting in a uniform movement of the structure. How to achieve: actuatable units are co-joined into a complete system and later into a much larger system of systems.	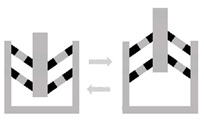	[72,73]
Compliant mechanisms	Description: mechanism in which the motion is not only governed by the geometry and mass distribution but also by the forces. How to achieve: a compliant mechanism gains its mobility from the deformation of its flexible member.	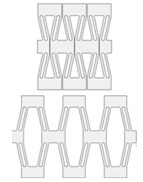	[74,75]
Non-topologically equivalent changing	Description: non-topologically equivalent changes occur when a construct is able to self-fill holes or self-repair cuts. How to achieve: exploiting the self-healing properties of certain polymers. The increase in temperature is a possible method to trigger non-topologically equivalent changes.	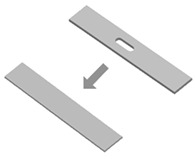	[76,77]

## Data Availability

No original data were reported in this review paper.
